# Optimal cerebral perfusion pressure via transcranial Doppler in TBI: application of robotic technology

**DOI:** 10.1007/s00701-018-3687-5

**Published:** 2018-09-29

**Authors:** Frederick A. Zeiler, Marek Czosnyka, Peter Smielewski

**Affiliations:** 1 Division of Anaesthesia, Addenbrooke’s Hospital, University of Cambridge, Cambridge, UK; 2 Department of Surgery, Rady Faculty of Health Sciences, University of Manitoba, Winnipeg, MB Canada; 3 Clinician Investigator Program, Rady Faculty of Health Sciences, University of Manitoba, Winnipeg, MB Canada; 4 Division of Neurosurgery, Department of Clinical Neurosciences, Addenbrooke’s Hospital, University of Cambridge, Cambridge, CB2 0QQ UK; 5 Institute of Electronic Systems, Warsaw University of Technology, Warsaw, Poland

**Keywords:** CPP optimum, Robotic transcranial Doppler, TCD, Traumatic brain injury, TBI

## Abstract

Individualized cerebral perfusion pressure (CPP) targets may be derived via assessing the minimum of the parabolic relationship between an index of cerebrovascular reactivity and CPP. This minimum is termed the optimal CPP (CPPopt), and literature suggests that the further away CPP is from CPPopt, the worse is clinical outcome in adult traumatic brain injury (TBI). Typically, CPPopt estimation is based on intracranial pressure (ICP)-derived cerebrovascular reactivity indices, given ICP is commonly measured and provides continuous long duration data streams. The goal of this study is to describe for the first time the application of robotic transcranial Doppler (TCD) and the feasibility of determining CPPopt based on TCD autoregulation indices.

**Electronic supplementary material:**

The online version of this article (10.1007/s00701-018-3687-5) contains supplementary material, which is available to authorized users.

## Introduction

Continuous monitoring of cerebrovascular reactivity in traumatic brain injury (TBI) is becoming increasingly common in the multi-modal monitoring (MMM) of critically ill patients [10, 11, 16]. To date, support for such monitoring has arisen within international consensus statements [10, 16]. Such support is centered on what is considered the “gold standard” continuous index, pressure reactivity index (PRx—correlation between intracranial pressure (ICP) and mean arterial pressure (MAP)) [11], given its association with global outcome in TBI and validation in experimental animal models against the lower limit of autoregulation [4, 23, 32]. Furthermore, literature supports the association of PRx-derived “personalized” cerebral perfusion pressure (CPP) targets (referred to as CPP optimum or CPPopt) and global outcome [19].

Numerous other continuous indices of cerebrovascular reactivity exist in the TBI literature [31, 32], derived from other invasive and non-invasive monitoring devices, including transcranial Doppler (TCD)-based measures. Indices based on TCD mean flow velocity (FVm) and systolic flow velocity (FVs) have been linked to global outcome in adult TBI [6, 22] and are known to be reasonably strongly associated with ICP-derived indices, such as PRx [30, 33].

However, despite the success with CPPopt determination using ICP indices [1, 19], it is unknown if TCD-based measures can be used to estimate this value, despite the strong association with ICP indices. This has not been explored in the past given TCD is typically limited by intermittent short duration recordings, given technical limitations. Various devices and probe holders have been designed for attempts at longer duration recordings, even with automated flow velocity detection algorithms [18, 28]. There are two main methods for assessment of cerebral physiology/structure that have been developed, including automated/semi-automated TCD methods (including robotic TCD) and phased-array-focused duplex methods [3, 20]. Such devices include but are not limited to those developed and/or distributed by Compumedics (Compumedics Germany GmbH, Sigen, Germany, https://www.compumedics.com.au/diagnostic-solution/transcranial-doppler/), Delica (Delica, Shenzhen, China, http://www.delicasz.com/html/en), and Pulse Medical (Pulse Medical Limited, Knaphill, UK, www.pulsemedical.co.uk/). However, with advancement in robotic TCD technology, it is possible to obtain relatively uninterrupted, extended duration recordings, allowing for the ability to assess CPPopt. In this article, we present a descriptive analysis of the first attempts at estimating CPPopt in critically ill adult TBI patients using extended duration recordings obtained from robotic TCD.

## Methods

### Patient population

This was a prospective observational study conducted over a 6-month period within our unit, during which we obtained a robotic TCD unit on trial. All patients suffered from moderate to severe TBI and were admitted to the neurosciences critical care unit (NCCU) at Addenbrooke’s Hospital, Cambridge, during the period of November 2017 to May 2018. Patients were intubated and sedated given the severity of their TBI. Invasive ICP monitoring was conducted in accordance with the Brain Trauma Foundation (BTF) guidelines. Therapeutic measures were directed at maintaining ICP less than 20 mmHg and CPP greater than 60 mmHg.

TCD is a part of standard intermittent cerebral monitoring within the NCCU. The application of the newer robotic TCD device were therefore in alignment with our usual care, negating the need for formal direct or proxy consent. All data related to patient admission demographics and high frequency digital signals from monitoring devices were collected in an entirely anonymous format, negating the need for formal consent, as in accordance with institutional research committee policies. Given limitations with the device, as outlined in a previous publication [34], not all patients admitted to the NCCU with TBI could be recorded with this device. In particular, the presence of decompressive craniectomy or unstable cervical spines precluded the application of the TCD device. In general, given the limitations outlined in our previous technology analysis [34], this led to our ability to apply the device in approximately 60–70% of all TBI patients admitted during the above described time period, with the majority being excluded secondary to decompressive craniectomy (primary or secondary) and having uncertain status of cervical spine stability. No patients were excluded after data sampling with the TCD device.

### Signal acquisition

Various signals were obtained through a combination of invasive and non-invasive methods. Arterial blood pressure (ABP) was obtained through either radial or femoral arterial lines connected to pressure transducers (Baxter Healthcare Corp. CardioVascular Group, Irvine, CA). ICP was acquired via an intra-parenchymal strain gauge probe (Codman ICP MicroSensor; Codman & Shurtleff Inc., Raynham, MA). Zeroing of the arterial line occurred at the level of the tragus during the course of this study.

**Fig. 1:**
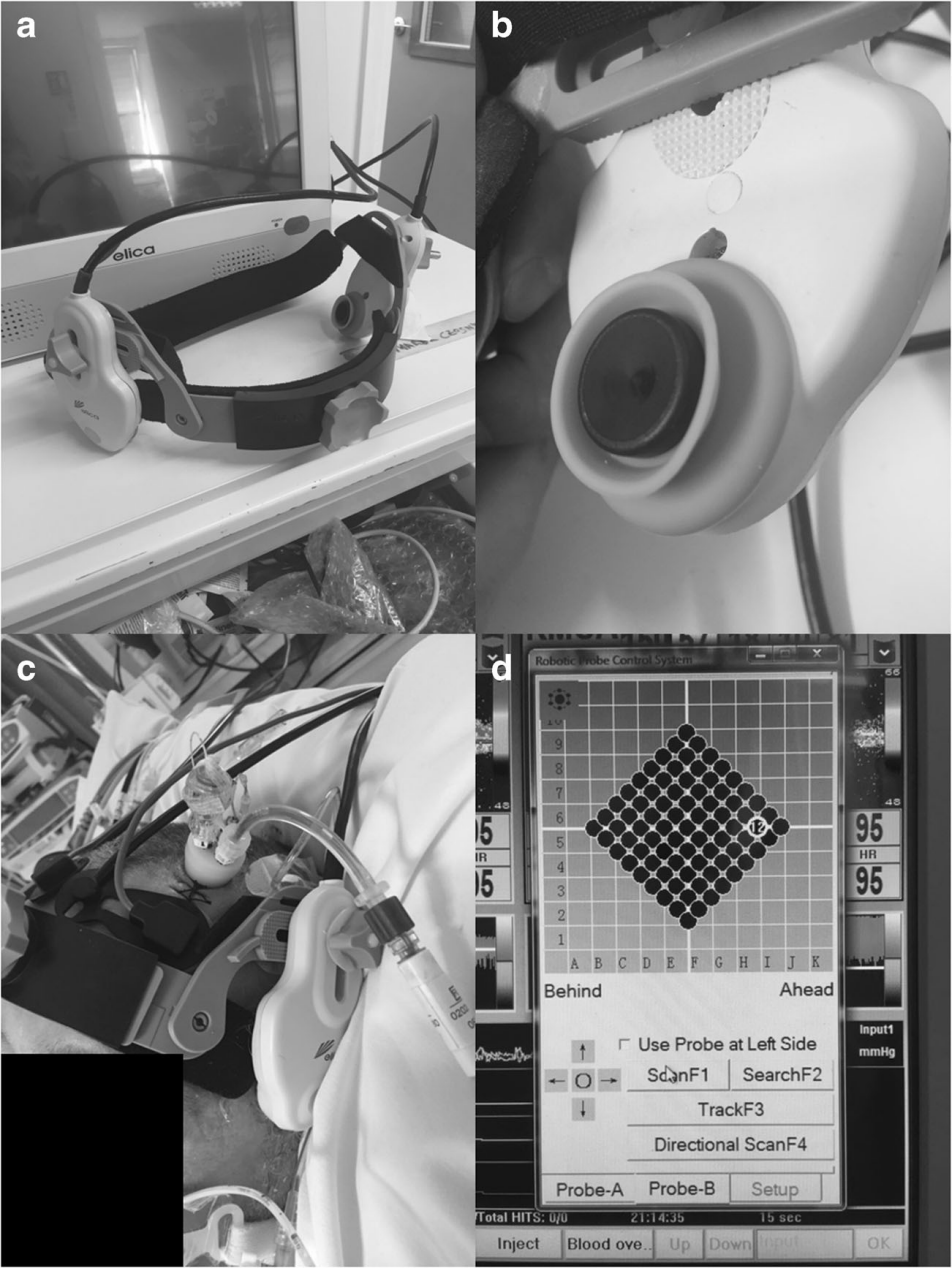
Example of recording set up for ICP and robotic TCD. ICP intracranial pressure, TCD transcranial Doppler. **a** Displays Delica robotic TCD headframe with two robotic drives mounted to a ratcheting headband system. **b** Displays close up image of TCD probe encased in robotic drive. **c** Example of set up for multi-modal monitoring with left frontal triple bolt (ICP, microdialysis and brain tissue oxygen monitoring), bifrontal near infrared spectroscopy, robotic TCD. **d** Delica EMS 9D TCD program display, showing automatic flow velocity sampling algorithm, producing a square grid of sampled insonation positions. The system insonates at multiple sites, finding the area with superior signal quality)

Finally, TCD assessment of MCA CBFV was conducted via a robotic TCD system, the Delica EMS 9D (Delica, Shenzhen, China, http://www.delicasz.com/html/en). This system allows for continuous extended duration recording of MCA CBFV, using 1.6 MHz robotically controlled TCD probes, with automated correction algorithms for probe shift. We aimed to record 3 to 4 h of continuous data from all devices simultaneously, given the previous work from our group on inter-index relationships focused on recording durations of only 0.5- to 1-h duration due to limitations of conventional TCD [22, 30]. Based on manufacturer specifications of the Delica robotic TCD system, the thermal index for the 1.6 MHz Doppler probes is less than 1.0, with the index less than 0.5 in most cases. As such, in keeping with the guidelines for adult TCD provided by the British Medical Ultrasound Society, the thermal index for the device is in the range acceptable for potentially “unlimited” TCD duration, while adhering to the principles of “as low as reasonably achievable” (ALARA) [5, 12, 13]. Thus, there were no concerns with tissue heating as a result of the extended duration recordings using this system. Brain temperature, local or global, was not recorded in this patient cohort. Figure 1 displays the robotic TCD device and set up for recording in critical ill TBI patients.

### Signal processing

Signals were recorded using digital data transfer, with sampling frequency of 100 Hz, using ICM+ software (Cambridge Enterprise Ltd., Cambridge, UK, http://icmplus.neurosurg.cam.ac.uk). Signal artifact was removed using a combination of manual and semi-automated methods within ICM+ prior to further processing or analysis.

Post-acquisition processing of the above signals was conducted using ICM+ software. CPP was determined using the formula: CPP = MAP − ICP. TCD signal was analyzed from the right side in the majority of the patients given right frontal placement of ICP monitors. Therefore, all continuous indices derived are based on mainly right-sided TCD recording. The only exception to this is when we were unable to obtain sufficient quality TCD signal on the right.

Systolic flow velocity (FVs) was determined by calculating the maximum flow velocity (FV) over a 1.5 s window, updated every second. Diastolic flow velocity (FVd) was calculated using the minimum FV over a 1.5 s window, updated every second. Mean flow velocity (FVm) was calculated using average FV over a 10 s window, updated every 10 s (i.e., without data overlap). Pulse amplitude of ICP (AMP) was determined by calculating the fundamental Fourier amplitude of the ICP pulse waveforms over a 10 s window, updated every 10 s.

Ten second moving averages (updated every 10 s to avoid data overlap) were calculated for all recorded signals: ICP, ABP (which produced MAP), CPP, FVm, FVs, andFVd. These non-overlapping 10-s moving average values allow focus on slow-wave fluctuations in signals by decimating the signal frequency to ~ 0.1 Hz.

Autoregulation indices were derived in a similar fashion across modalities; an example is provided for PRx: A moving Pearson correlation coefficient was calculated between ICP and MAP using 30 consecutive 10 s windows (i.e., five minutes of data), updated every minute. Details on each index calculation can be found in Table 1.

**Table 1:** Autoregulation indices and calculation methods

Index	Signals correlated	Signal averaging (sec)	Pearson correlation coefficient calculation window (min)	Index calculation update frequency (sec)
PRx	ICP and MAP	10	5	60
PAx	AMP and MAP	10	5	60
RAC	AMP and CPP	10	5	60
Mx	FVm and CPP	10	5	60
Mx_a	FVm and MAP	10	5	60
Sx	FVs and CPP	10	5	60
Sx_a	FVs and MAP	10	5	60

*AMP*, pulse amplitude of ICP; *CPP*, cerebral perfusion pressure; *FVd*, diastolic flow velocity; *FVm*, mean flow velocity; *FVs*, systolic flow velocity; *ICP*, intracranial pressure; *MAP*, mean arterial pressure, *min*, minute; *sec*, seconds; *Mx*, mean flow index (correlation between FVm and CPP); *Mx_a*, Mx based on MAP (correlation between FVm and MAP); *PAx*, pulse amplitude index (correlation between AMP and MAP); *PRx*, pressure reactivity index (correlation between ICP and MAP); *Sx*, systolic flow index (correlation between FVs and CPP); *Sx_a*, Sx based on MAP (correlation between FVs and MAP); *RAC*, correlation (R) between AMP (A) and CPP (C)

Data for this analysis were provided in the form of a minute by minute time trends of the parameters of interest for each patient. This was extracted from ICM+ in to comma separated values (CSV) datasets, which were collated into one continuous data sheet (compiled from all patients).

### Descriptive analysis

R statistical software (R Core Team (2016). R: A language and environment for statistical computing. R Foundation for Statistical Computing, Vienna, Austria. URL https://www.R-project.org/) was utilized for post processing of ICM+ data outputs, producing population wide binned error bar plots of various cerebrovascular reactivity indices versus CPP, to highlight the population-based parabolic relationships between both ICP and TCD indices with CPP. Mean index values were calculated across 5 mmHg bins of CPP for the entire population.

Finally, ICM+ was used to produce individual patient CPPopt plots for the purpose of examining feasibility of TCD-based CPPopt estimation in patients with extended duration uninterrupted recordings (i.e., ~ 4-h duration).

## Results

### Patient demographics

During the 6-month trial period for the robotic TCD device, we were able to record 20 critically ill adult TBI patients. Due to limitations imposed by the robotic probe design there are certain contra-indications that prevented its application in some critically ill TBI patients. These include decompressive craniectomy, extensive soft tissue damage to the scalp, and unstable cervical spine (including those with uncertain cervical spine status). Patient demographics can be seen in Appendix A. Overall, the mean age was 42.6 ± 17.6 years, with 12 patients being male, and a median admission Glasgow Coma Scale score of 7 (inter-quartile range (IQR): 5 to 8). The mean duration of TCD recording was 224.8 ± 40.2 min. Though it is acknowledged, not all recordings were completely uninterrupted, given the need for urgent scans and bedside nursing requests for probe removal in 10 patients.

### CPP optimum—patient example

**Fig. 2:**
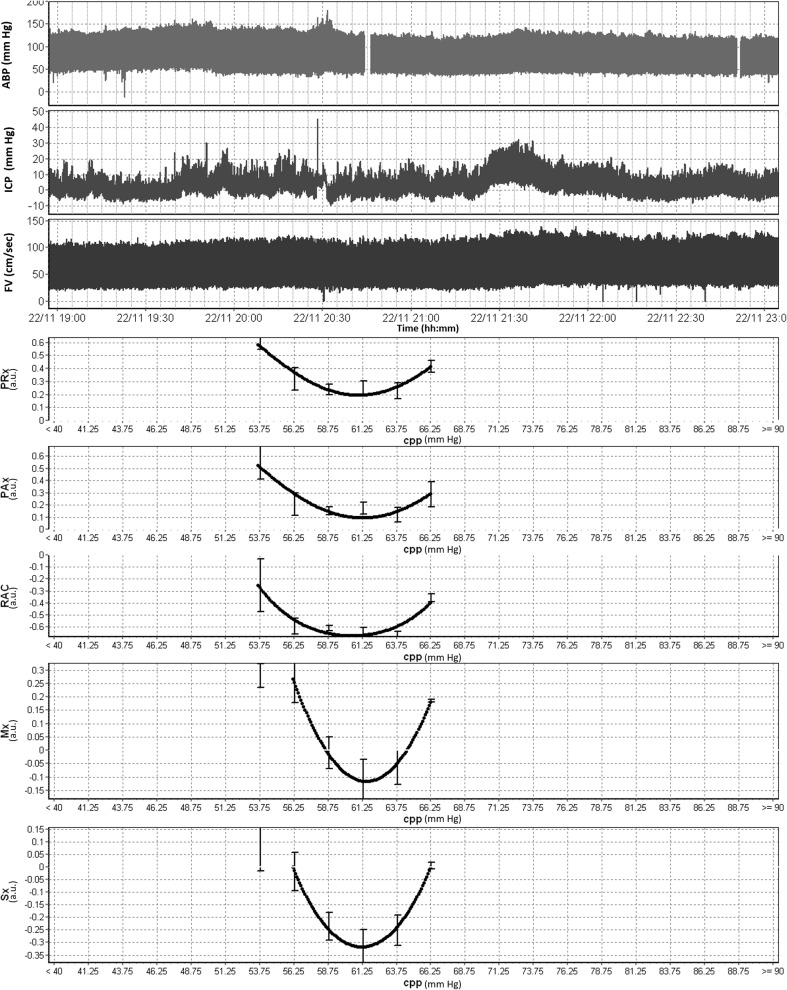
Patient example of CPPopt estimation using ICP- and TCD-based indices. ABP, arterial blood pressure; AMP, pulse amplitude of ICP; a.u., arbitrary units; cm, centimeters; CPP, cerebral perfusion pressure; FV, flow velocity; FVm, mean flow velocity; FVs, systolic flow velocity; hh:mm, hours:minutes; ICP, intracranial pressure; MAP, mean arterial pressure; mmHg, millimeters of mercury; Mx, mean flow index (correlation between FVm and CPP); PAx, pulse amplitude index (correlation between AMP and MAP); PRx, pressure reactivity index (correlation between ICP and MAP); RAC, correlation between AMP and CPP; sec, second; Sx, systolic flow index (correlation between FVs and CPP). *Top 3 panels display raw signal for ABP, ICP and FV over 4 h window, demonstrating stable continuous TCD recordings. The bottom 5 panels display CPPopt plots for PRx, PAx, RAC, Mx, and Sx

In five patients with the longest duration of uninterrupted recordings (i.e., those reaching ~ 4 h in duration), it was possible to estimate CPPopt via plotting mean cerebrovascular reactivity index versus CPP. Figure 2 displays an example of one such recording, where CPPopt could be estimated using both ICP indices (i.e., PRx, PAx, and RAC, see Table 1 for calculations) as well as TCD indices (Sx and Mx, see Table 1 for calculations), using 2.5 mmHg bins of CPP. Similar CPPopt plots for the MAP-based Sx_a and Mx_a (see Table 1 for calculations) can be found in Appendix B.

### Population wide error Bar plots

**Fig. 3:**
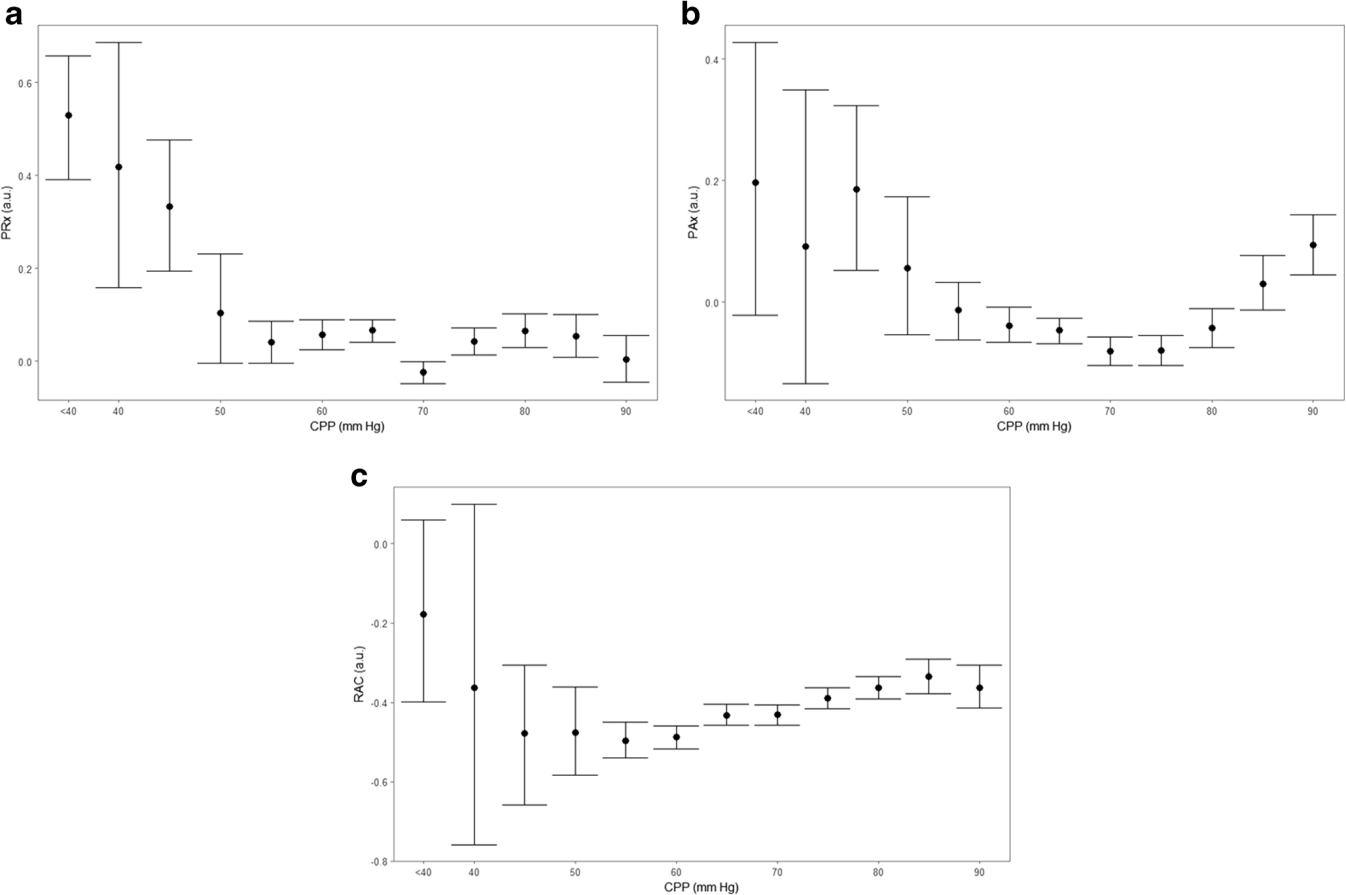
Population wide error bar plots—ICP-derived indices. AMP, pulse amplitude of ICP; a.u., arbitrary units; CPP, cerebral perfusion pressure; ICP, intracranial pressure; MAP, mean arterial pressure; mmHg, millimeters of mercury; PAx, pulse amplitude index (correlation between AMP and MAP); PRx, pressure reactivity index (correlation between ICP and MAP); RAC, correlation between AMP and CPP. **a** PRx vs. CPP. **b** PAx vs. CPP. **c** RAC vs. CPP. Note: the lack of distinct parabolic relationship in the PRx plot likely stems for the low mean ICP (i.e., ~ 11 mmHg) for the population over the course of the 3- to 4-h recordings. PAx and RAC display superior parabolic relationships with CPP likely secondary to the slow-wave fluctuation between AMP and MAP/CPP being easier to assess than that of ICP and MAP in the setting of persistently low ICP values

**Fig. 4:**
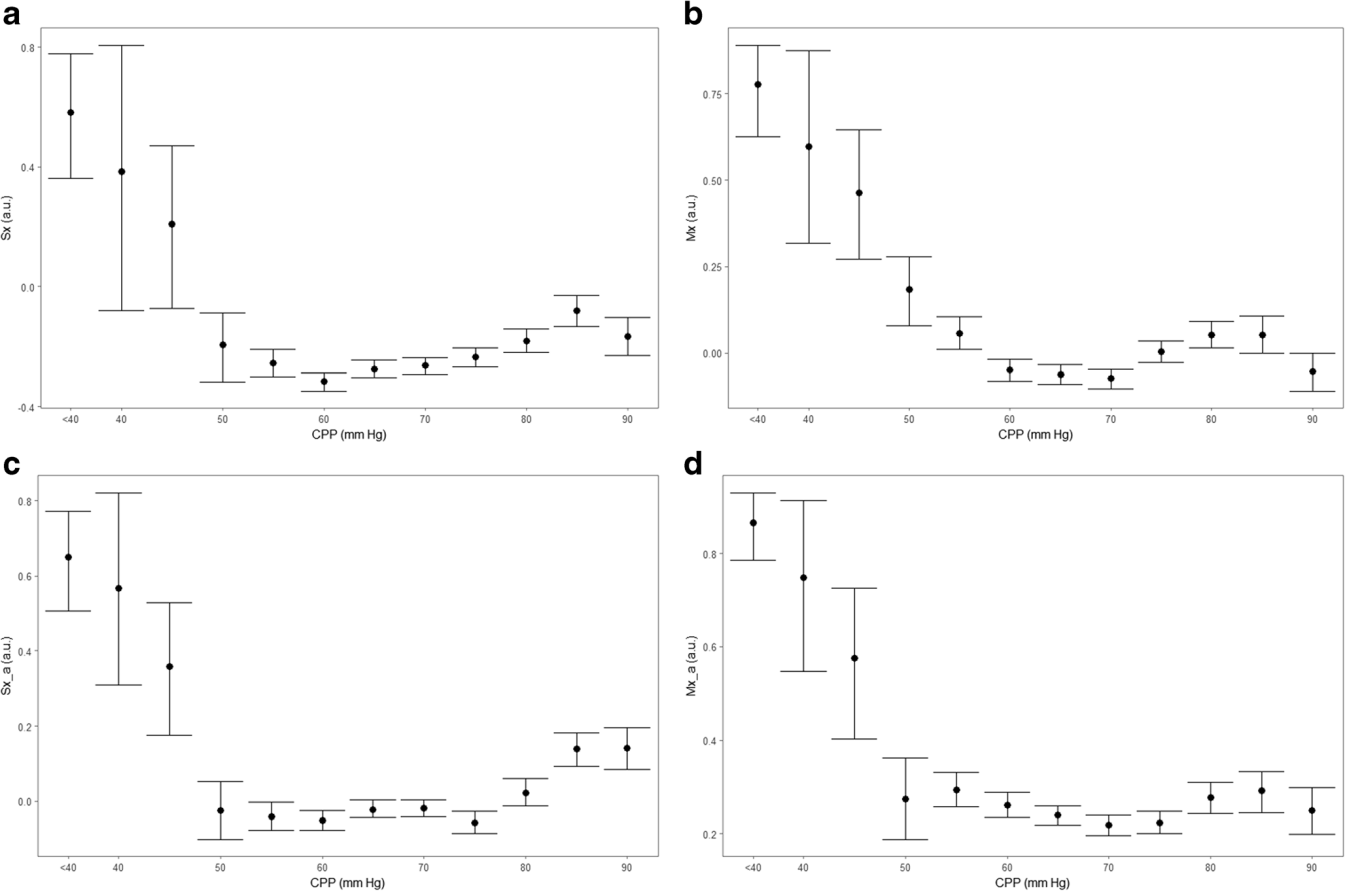
Population wide error bar plots—TCD-based indices. a.u., arbitrary units; CPP, cerebral perfusion pressure; FVm, mean flow velocity; FVs, systolic flow velocity; ICP, intracranial pressure; MAP, mean arterial pressure; mmHg, millimeters of mercury; Mx, mean flow index (correlation between FVm and CPP); Mx_a, MAP-based mean flow index (correlation between FVm and MAP); Sx, systolic flow index (correlation between FVs and CPP); Sx_a, MAP-based systolic flow index (correlation between FVs and MAP). **a** Sx vs. CPP. **b** Mx vs. CPP. **c**: Sx_a vs. CPP. **d** Mx_a vs. CPP

Using the entire 20 patients recorded, we produced various binned error bar plots of ICP and TCD cerebrovascular reactivity indices across 5 mmHg bins of CPP. Figure 3 displays the error bar plots for the ICP-based indices, while Fig. 4 displays the error bar plots for the TCD-based indices. As seen from the plots, at the population level, the TCD-derived indices display a parabolic relationship with CPP, suggesting the feasibility of CPPopt determinations using these TCD measures.

## Discussion

Our simple descriptive analysis of CPPopt estimation using extended duration TCD recordings obtained from robotic TCD highlights what is possible with advanced TCD technology when applied to monitoring critically ill TBI patients. We have demonstrated the feasibility of TCD-based CPPopt estimation, and that these TCD-based cerebrovascular reactivity indices do indeed have a parabolic relationship with CPP, as seen classically with ICP-based indices [1, 2, 14, 17], though it must be acknowledged that the duration of recordings reported within this analysis allowed for a single spot assessment of CPPopt using robotic TCD. Thus, in its current state, this allows for a limited “spot” check of a patient's individualized CPPopt, unlike current invasively derived CPPopt which provides a continuous updating value more applicable for routine clinical monitoring. The goal of this study was not to demonstrate equivalency or superiority of TCD-based CPPopt over existing invasively derived versions, but to display some potential uses of this ever evolving robotic TCD technology. As such, no direct comparison between such CPPopt values is provided in this preliminary pilot analysis, as it would be improper to do so based on the limited data acquired. It must emphasized, that this is just a pilot analysis and requires much further work in larger populations with extended duration TCD recordings, in order to determine the role for TCD-based CPPopt and whether these measures relate to clinically relevant outcomes in adult TBI. Furthermore, comparison of TCD-based CPPopt and ICP-derived CPPopt needs to occur in a large cohort of patients with long TCD recordings, in order to assess if the temporal profiles of these two CPPopt measures are the same, or at least related over time.

Another important aspect is that CPPopt is not a validated CPP target in TBI, as of yet. The concept of CPPopt directed therapy in moderate/severe TBI has not been validated in large prospective randomized control trials. However, numerous retrospective studies have been published assessing the relationship between CPP values outside of the CPPopt range, and global outcome [1, 19, 24]. Having CPP below CPPopt has been documented to be associated with poor global outcome, mortality, and morbidity, in adult TBI [14, 19, 24]. One may also be concerned that targeting CPPopt will lead to a “drift” in CPP targets to higher levels, above and beyond the currently recommended BTF guidelines [9]. However, the association with CPP values above CPPopt is quite unclear. One retrospective study demonstrated a potential association with increase severe disability, though this has not been replicated [1]. Other studies have failed to produce statistically significant associations between CPP above CPPopt and patient global outcome [19]. Furthermore, recent work suggests that CPP values above CPPopt may not even be linked to cardiopulmonary complications, such as acute respiratory distress syndrome (ARDS) [25, 29], which has long been the concern of having CPP values above the defined BTF guidelines. All of this uncertainty has led to the phase II randomized control trial of CPPopt versus BTF-based CPP therapy in adult TBI. It will not be until the completion of the planned phase III trial that we will be able to potentially comment on the above outlined uncertainties with CPPopt, and clarify the role of CPPopt therapy in adult TBI.

Given the recorded population consisted of only a small pilot group during the trial period for this device, the ability to extrapolate the results of this study to other populations is limited. This small population is secondary to current technology limitations (as described in the results) and ongoing user presence during recordings. Thus, with this technology, TCD becomes less involved, however, it is still somewhat labor intensive compared to other monitoring devices employed in critically ill TBI patients.

One may question the use of TCD in the presence of existing ICP monitoring, particularly when it comes to assessing autoregulation/vascular reactivity. However, ICP, as used in PRx calculations, has some conceptual problems when assessing cerebrovascular reactivity. First, ICP is considered a surrogate for changes in pulsatile cerebral blood volume (CBV) and relies on the pressure-volume relationship. TCD is a close direct measure to cerebral blood flow (CBF) and likely provides more useful information for assessment of autoregulation. Second, PRx does not measure autoregulation directly; it is an index of vasodilation/constriction in response to CPP changes, whereas TCD flow velocity reflects changes in CBF, which is what we are really interested in. Finally, ICP is a regional measure that is approximated to global compartmental reading. With TCD, one can obtain territory specific information and assess hemispheric asymmetry.

The concept of TCD-based CPPopt may be of question as well, since CPP currently requires ICP to obtain. However, efforts are ongoing for non-invasive TCD-based methods of CPP measurement [7, 8, 15, 21, 26, 27]. With the acquisition of extended duration uninterrupted robotic TCD recordings, the potential to have a more continuous TCD-based CPP measure is feasible, though further research is required. Combining TCD measured CPP with non-invasive TCD cerebrovascular reactivity indices, such as Sx_a or Mx_a (acquired using TCD in conjunction with non-invasive continuous ABP), one could in theory obtain CPPopt through an entirely non-invasive means. Again, much further work in this area is required. However, as robotic TCD technology continues to improve, we will see longer duration of uninterrupted recordings, and hopefully a move to less invasive monitoring in TBI.

## Conclusions

With the application of robotic TCD technology, it is possible to obtain extended duration recordings in critically ill TBI patients, allowing for the approximation of CPPopt using TCD-based cerebrovascular reactivity indices. As robotic TCD technology continues to advance, further long-term recording is becoming possible, with minimal user input, allowing for inclusion of continuous, uninterrupted, TCD monitoring into the standard set of neuromonitoring modalities in TBI patients.

## Electronic supplementary material

ESM 1 (DOCX 13 kb)

ESM 2 (DOCX 111 kb)
