# Supplementary material for: Optimal cerebral perfusion pressure via transcranial Doppler in TBI: application of robotic technology
Source: Acta Neurochir (Wien). 2018 Sep 29;160(11):2149–57. doi: 10.1007/s00701-018-3687-5 (PMC6209007; doi:10.1007/s00701-018-3687-5)
Supplement: Supplementary file 1 — (DOCX 13 kb) [file 701_2018_3687_MOESM1_ESM.docx]

Appendix A: Patient Demographics

|  | | **Mean/Median (+/- SD; IQR)** |
| --- | --- | --- |
| ***N*** | | 20 |
| ***Age (years)*** | | 42.6 (17.6) |
| ***Sex*** | ***Male*** | 12 |
|  | ***Female*** | 8 |
| ***Admission GCS – Total*** | | 7 (5 to 8) |
| ***Admission GCS – Motor*** | | 4 (3 to 5) |
| ***Pupil Reactivity*** | ***Bilaterally Reactive*** | 14 |
|  | ***Unilateral Reactive*** | 5 |
|  | ***Bilaterally Unreactive*** | 1 |
| ***MAP (mm Hg)*** | | 86.1 (12.6) |
| ***ICP (mm Hg)*** | | 11.1 (6.4) |
| ***CPP (mm Hg)*** | | 75.0 (9.9) |
| ***PRx (a.u.)*** | | 0.040 (0.206) |
| ***PAx (a.u.)*** | | -0.053 (0.127) |
| ***RAC (a.u.)*** | | -0.420 (0.181) |
| ***Sx (a.u.)*** | | -0.225 (0.276) |
| ***Sx_a (a.u.)*** | | -0.003 (0.212) |
| ***Mx (a.u.)*** | | -0.11 (0.276) |
| ***Mx_a (a.u)*** | | 0.247 (0.171) |

AMP = pulse amplitude of ICP, a.u. = arbitrary units, CPP = cerebral perfusion pressure, FVm = mean flow velocity, FVs = systolic flow velocity, GCS = Glasgow Coma Scale, ICP = intra-cranial pressure, IQR = inter-quartile range, MAP = mean arterial pressure, Mx = mean flow index (correlation between FVm and CPP), Mx_a = MAP based mean flow index (correlation between FVm and MAP), N = number of patients, PAx = pulse amplitude index (correlation between AMP and MAP), PRx = pressure reactivity index (correlation between ICP and MAP), RAC = correlation between AMP and CPP, SD = standard deviation, Sx = systolic flow index (correlation between FVs and CPP), Sx_a = MAP based systolic flow index (correlation between FVs and MAP). *Mean values were calculated using grand mean values of physiologic variables per patient, calculated the population mean off of these grand mean patient values.
