# Supplementary material for: Optimal cerebral perfusion pressure via transcranial Doppler in TBI: application of robotic technology
Source: Acta Neurochir (Wien). 2018 Sep 29;160(11):2149–57. doi: 10.1007/s00701-018-3687-5 (PMC6209007; doi:10.1007/s00701-018-3687-5)
Supplement: Supplementary file 2 — (DOCX 111 kb) [file 701_2018_3687_MOESM2_ESM.docx]

Appendix B: Patient Example – CPPopt Curves for MAP Based Sx_a and Mx_a


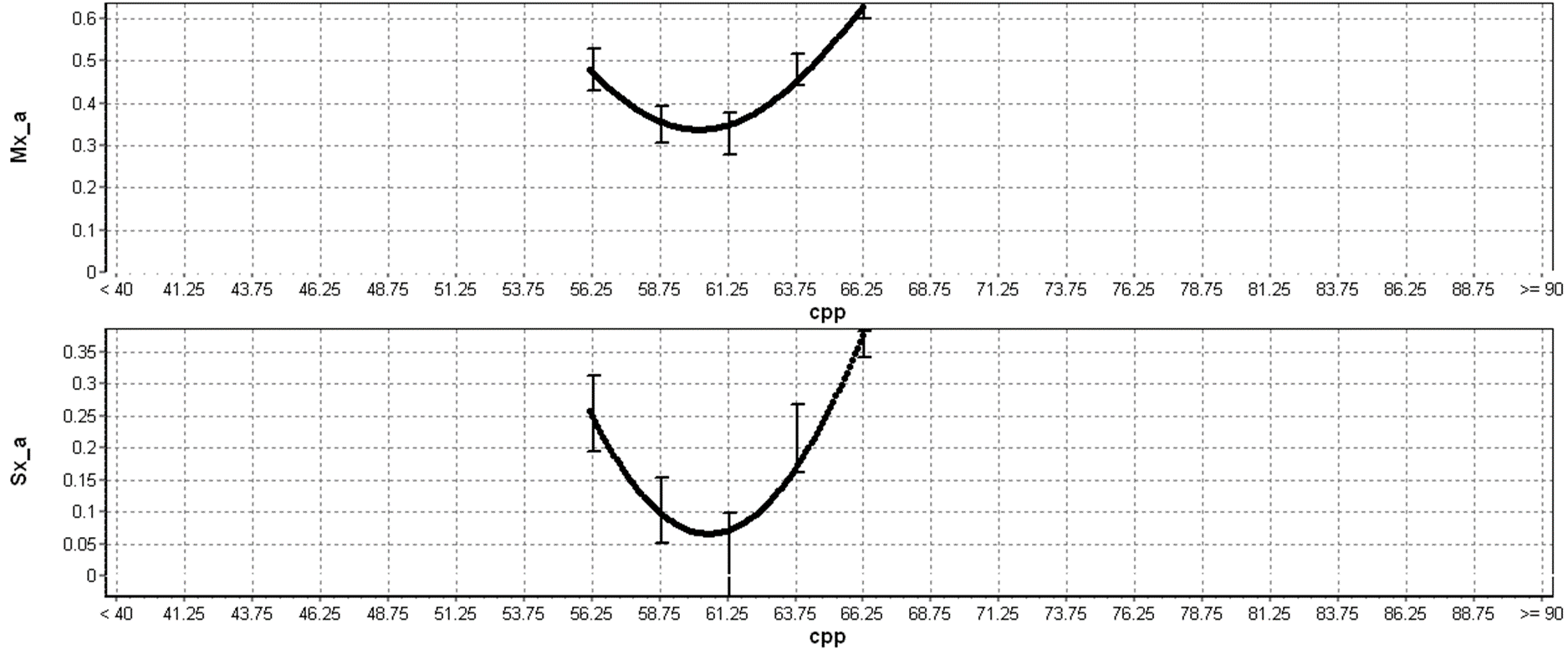


a.u. = arbitrary units, cm = centimeters, CPP = cerebral perfusion pressure, FVm = mean flow velocity, FVs = systolic flow velocity, MAP = mean arterial pressure, Mx_a = MAP based mean flow index (correlation between FVm and MAP), Sx_a = MAP based systolic flow index (correlation between FVs and MAP).
